# An Exochitinase with *N*-Acetyl-β-Glucosaminidase-Like Activity from Shrimp Head Conversion by *Streptomyces speibonae* and Its Application in Hydrolyzing β-Chitin Powder to Produce *N*-Acetyl-d-Glucosamine

**DOI:** 10.3390/polym11101600

**Published:** 2019-09-30

**Authors:** Thi Ngoc Tran, Chien Thang Doan, Minh Trung Nguyen, Van Bon Nguyen, Thi Phuong Khanh Vo, Anh Dzung Nguyen, San-Lang Wang

**Affiliations:** 1 Department of Chemical and Materials Engineering, Tamkang University, New Taipei City 25137, Taiwan; tranngoctnu@gmail.com; 2 Department of Chemistry, Tamkang University, New Taipei City 25137, Taiwan; doanthng@gmail.com; 3 Doctoral Program in Applied Sciences, College of Science, Tamkang University, New Taipei City 25137, Taiwan; 4 Department of Science and Technology, Tay Nguyen University, Buon Ma Thuot 630000, Vietnam; 5 Institute of Biotechnology and Environment, Tay Nguyen University, Buon Ma Thuot 630000, Vietnam; nadzungtaynguyenuni@yahoo.com.vn; 6 Life Science Development Center, Tamkang University, New Taipei City 25137, Taiwan

**Keywords:** exochitinase, β-chitin powder, *N*-acetyl-β-glucosaminidase, shrimp heads, *Streptomyces speibonae*

## Abstract

Marine chitinous byproducts possess significant applications in many fields. In this research, different kinds of fishery chitin-containing byproducts from shrimp (shrimp head powder (SHP) and demineralized shrimp shell powder), crab (demineralized crab shell powder), as well as squid (squid pen powder) were used to provide both carbon and nitrogen (C/N) nutrients for the production of an exochitinase via *Streptomyces speibonae* TKU048, a chitinolytic bacterium isolated from Taiwanese soils. *S. speibonae* TKU048 expressed the highest exochitinase productivity (45.668 U/mL) on 1.5% SHP-containing medium at 37 °C for 2 days. Molecular weight determination analysis basing on polyacrylamide gel electrophoresis revealed the mass of TKU048 exochitinase was approximately 21 kDa. The characterized exochitinase expressed some interesting properties, for example acidic pH optima (pH 3 and pH 5–7) and a higher temperature optimum (60 °C). Furthermore, the main hydrolysis mechanism of TKU048 exochitinase was *N*-acetyl-β-glucosaminidase-like activity; its most suitable substrate was β-chitin powder. The hydrolysis experiment revealed that TKU048 exochitinase was efficient in the cleavage of β-chitin powder, thereby releasing *N*-acetyl-d-glucosamine (GlcNAc, monomer unit of chitin structure) as the major product with 0.335 mg/mL of GlcNAc concentration and a yield of 73.64% after 96 h of incubation time. Thus, TKU048 exochitinase may have potential in GlcNAc production due to its *N*-acetyl-β-glucosaminidase-like activity.

## 1. Introduction

Chitin is a straight-chain polymer of *N*-acetyl-d-glucosamine (GlcNAc) unit with β-1,4 linkage, which is a very common polysaccharide in the world, second only to cellulose. By expressing various bioactivities, chitin and its derivatives are of interest to numerous researchers [1,2,3,4,5,6,7,8]. Until now, chitin-containing materials from fishery byproducts (shells from crab or shrimp, or squid pens) are the most important sources for chitin production. However, those chitinous materials also contain a high amount of proteins as well as minerals [9,10]. Consequently, strong alkali and acids are used to remove the protein and mineral salts from these resources to produce chitin. As a result, these chemical procedures encounter several drawbacks when these chemical procedures are applied, such as the release of alkaline wastewater containing a high concentration of protein [11]. In green applications*,* those chitin-containing byproducts could also be used as the nutrition sources for microorganism bioconversion to produce numerous bioactive compounds, for instance, proteases [9,11,12], chitinases/chitosanases [2,4,13,14,15,16,17,18], α-glucosidase inhibitors [19,20,21,22,23,24,25], exopolysaccharide [26,27,28], tyrosinase inhibitors [29,30], or chitin [1,31,32,33].

Bacterial strains, which include *Bacillus* [4,24,34], *Paenibacillus* [11,20,35], *Serratia* [36], and *Streptomyces* [2], have been reported as the primary sources for chitinase production. Among these, chitinase from various *Streptomyces* strains has been investigated [37,38,39,40,41]; however, most of those researches used colloidal chitin (CC) as the source of carbon and nitrogen (C/N) for chitinolytic enzyme production. In addition, there are few reports on chitinase production from *Streptomyces* using squid pens, shrimp shells, or shrimp heads as the main source of C/N [2]. Based on the above, it is interesting to investigate the application of shrimp heads for the production of chitinase via *Streptomyces* bioconversion.

GlcNAc, the monomeric unit of chitin, has been found to exhibit many bioactivities that have been widely applied in food, pharmaceutical, biomedical, and chemical industries [42,43,44]. Therefore, the hydrolysis of chitin to produce GlcNAc has been explored [43]. Due to its chitin hydrolysis ability, chitinase may be an efficient tool in GlcNAc production from chitin. Chitinases (EC.3.2.14) can be divided into two groups: exochitinase and endochitinase. While endochitinase randomly cleaves chitin at internal sites, exochitinase (divided into two subcategories: chitobiosidase and *N*-acetyl-β-glucosaminidase) acts at the end point of chitin oligosaccharides to liberate (GlcNAc)_2_ (chitobiosidase) or GlcNAc (*N*-acetyl-β-glucosaminidase) [43]. Thus, the finding of a chitinolytic enzyme with *N*-acetyl-β-glucosaminidase-like activity could prove promising in regard to its potential for the production of GlcNAc by the enzymatic method.

In this research, an exochitinase-producing *Streptomyces speibonae* TKU048, was isolated in Northern Taiwan using squid pen powder (SPP) as the sole source of C/N. The optimal conditions for exochitinase production on different kinds of fishery chitin-containing byproducts from shrimp (shrimp head powder (SHP) and demineralized shrimp shell powder (deSSP)), crab (demineralized crab shell powder (deCSP)), as well as squid (squid pen powder (SPP)) and the enzyme characteristics have been investigated. Furthermore, TKU048 exochitinase has been evaluated in relation to GlcNAc production by using β-chitin powder as substrate.

## 2. Materials and Methods

### 2.1. Materials

Chitinous byproducts were obtained from Fwu-Sow Industry (Taichun, Taiwan) (for shrimp head powder (SHP)) and Shin-Ma Frozen Food Co. (I-Lan, Taiwan) (for crab shells, shrimp shells, and squid pens) [2]. Strong acid was applied to remove the mineral components in crab shell and shrimp shell to produce demineralized shrimp shell and demineralized crab shell [20]. 3,5-Dinitrosalicylic acid (DNS), *p*-nitrophenol (*p*NP), *p*-nitrophenyl-*N*-acetyl-β-d-glucosaminide (*p*NPg), and *N*-acetyl-d-glucosamine used for determining chitinase activity were obtained from Sigma-Aldrich Corp. (Germany). The resin for ion-exchange chromatography was purchased from BioRad (Hercules, CA, USA). Column KW-802.5 and KS-802 were obtained from Showa Denko K. K (Tokyo, Japan). Other chemicals used in this study were the highest quality available.

### 2.2. Screening of Exochitinase-Producing Bacterium

The isolation was conducted on the medium containing squid pen powder (SPP, 1% *w*/*v*), MgSO_4_·7H_2_O (0.05% *w*/*v*), K_2_HPO_4_ (0.1% *w*/*v*), and agar (2% *w*/*v*). Firstly, 1 g of each soil sample, obtained from arable lands in Northern Taiwan, was gently shaken with 100 mL of sterile saline for 5 min. The suspension was then serially diluted until a 10^6^-fold dilution was obtained. One hundred microliters of the final dilution were spread over a Petri dish containing isolation medium. Inoculated Petri dishes were incubated at 37 °C for 24 h to get single colonies of bacteria. The pure bacterial strains were then taken from the single colonies by streaking method. Following this, each isolated strain was transferred to liquid media (1% SPP, 0.05% MgSO_4_·7H_2_O, and 0.1% K_2_HPO_4_) and cultivated for 3 days at 37 °C and 150 rpm. The culture of each bacterial strains was centrifuged again at 12,000× *g* (Universal 320, Hettich Zentrifugen, Tuttlingen, Germany) for 10 min to collect the supernatant, which was tested for exochitinase and chitinase activities. The bacterial strain which possessed the highest exochitinase activity was named as TKU048 and selected for further experiments. DNA sequencing, as well as biochemical and morphological methods were used to verify the identity of the TKU048 strain.

### 2.3. Enzyme Activity Assays

#### 2.3.1. Exochitinase Activity Assay

Determination of exochitinase activity was conducted following a previously described method [2]. Briefly, 50 µL of sample (containing exochitinase) was transferred to a tube containing 500 µL of sodium acetate buffer (50 mM, pH 5.8) and 100 µL of *p*NPg (1 mg/mL). The tube was immediately incubated for 30 min at 37 °C. Sodium carbonate–bicarbonate buffer (325 µL) was added to the reaction solution to eliminate exochitinase activity and introduce *p*NP coloration, which was measured by a spectrophotometer at 420 nm. The amount of exochitinase which catalyzed the hydrolysis reaction of *p*NPg to liberate 1 µmol of *p*NP in 1 min was defined as one unit (U) of enzyme activity.

#### 2.3.2. Chitinase Activity Assay

Chitinase activity assay was performed by the method of Doan et al. using colloidal chitin as substrate and *N*-acetyl-glucosamine as reference. The amount of chitinase which catalyzed the hydrolysis of colloidal chitin to liberate 1 µmol of *N*-acetyl-glucosamine in 1 min, was defined as one unit (U) of enzyme activity.

### 2.4. Culture Conditions for Exochitinase Production

One gram of each different fishery byproduct, including deCSP, deSSP, SHP, and SPP, was added to a glass Erlenmeyer flask (250 mL) containing 100 mL of basal salt medium to provide the carbon and nitrogen (C/N) nutrients for the growth and exochitinase production of *S*. *speibonae* TKU048. The culture was started by adding 1% (v/v) of stock solution of *S*. *speibonae* TKU048 and maintained under the following conditions: 37 °C incubation temperature and 150 rpm of agitation. An aliquot of culture (1 mL) was withdrawn every 24 h for testing exochitinase activity. After finding the best source of carbon and nitrogen for enzyme production, the optimization of culture conditions was further carried out for other parameters, including amount of C/N source (0.5%–2%, *w*/*v*), incubation temperature (30–50 °C), agitation speed (0–200 rpm), and initial pH (5–8).

### 2.5. Isolation of TKU048 Exochitinase

*S*. *speibonae* TKU048 was cultured as described above. One liter of culture supernatant was used for isolating TKU048 exochitinase. Further isolation steps included protein concentration by (NH_4_)_2_SO_4_ (80% saturation), Macro-Prep High Q chromatography, and KW-802.5 size-exclusion chromatography. These steps have been described in detail in a previous report [2]. The molecular weight of the TKU048 exochitinase was determined by SDS-PAGE analysis [2].

### 2.6. Effects of Temperature and pH on Enzyme Activities

The optimal temperature of TKU048 exochitinase was investigated by incubating the mixtures of enzyme and *p*NPg at different temperature points (from 20 to 100 °C) for 30 min. Meanwhile, the residual activity of enzyme solutions, which were pretreated in different temperatures for 30 min, was used to explore the thermal stability of TKU048 exochitinase. The optimal pH and pH stability of TKU048 exochitinase were carried out following the method of Tran et al. [2].

### 2.7. Effects of Ion Metals on Enzyme Activity

A similar amount of TKU048 exochitinase solutions were incubated with each of different ion metals (FeCl_2_, CaCl_2_, BaCl_2_, NaCl, MgCl_2_, ZnCl_2_, and CuCl_2_) and a metalloenzyme inhibitor (EDTA) at 20 °C for 30 min. The residual activity of TKU048 exochitinase was then measured according to the exochitinase activity assay, as described above.

### 2.8. Substrate Specificity Determination

Various substrates were used to explore the substrate specificity of TKU048 exochitinase, including *p*NPg, dextran (from *Leuconostoc* spp.), β-chitin powder (βCP), water-soluble chitosan (WSC, 60% of degree of deacetylation, DD), colloidal α-chitin (CC, from shrimp shell), cellulose, α-chitin powder (αCP), and colloidal chitosan (CCO, from shrimp shell, 60% of DD).

### 2.9. Hydrolysis Mechanism

To investigate the hydrolysis mechanism of TKU048 exochitinase, chitin oligosaccharides with degree of polymerizations (DP) 2–6 were used as the substrates. Five hundred microliters of substrate solution (0.5 mg/mL) was mixed with 500 µL enzyme solution (2 U, approximately) in the glass tubes. The reactions were subsequently carried out at 50 °C using a water bath. After every 20 min, 100 µL of each solution was withdrawn for analysis by HPLC method (described below).

### 2.10. HPLC Analysis

The chitin oligosaccharides and β-chitin powder hydrolysates, which were produced from the hydrolysis reaction catalyzed by TKU048 exochitinase, were analyzed by a Hitachi Chromaster HPLC system (column, KS-802; flow rate, 0.6 mL/min; column temperature, 80 °C; mobile phase, H_2_O; ultraviolet detection wavelength, 205 nm; volume of sample, 20 µL). To detect hydrolysis products, a series of chitin oligosaccharides (DP from 1 to 6) was used as a reference.

## 3. Results and Discussion

### 3.1. Screening of an Exochitinase-Producing Bacterium

More than 50 chitinolytic microorganisms from arable lands in Northern Taiwan were isolated on the media containing squid pen powder [2]. For producing chitinase activity, these strains were cultivated on liquid medium containing 1% SPP and mineral salts (0.05% MgSO_4_ and 0.1% K_2_HPO_4_) for 3 days under the following conditions: initial pH 7.2, 150 rpm, and 37 °C. Among them, the exochitinase activity of strain TKU048 culture revealed the highest value (4.285 U/mL). This strain was named as *Streptomyces speibonae* according to the results of 16S rRNA sequences as well as morphological and biochemical studies. So far, *Streptomyces* along with *Bacillus*, *Paenibacillus*, *Serratia*, and *Aspergillus* are the primary microbial strains producing chitinase. However, there are only a few reports on the production of exochitinase from *Streptomyces*, including *S. olivaceoviridis* [45], *S. thermocarboxydus* TKU045 [2], and *S. lividans* pCHIO12 [46]. Additionally, to our best knowledge, there are no reports on the production of exochitinase from *S. speibonae* species. Therefore, the discovery of exochitinase production in *S. speibonae* TKU048 is of interest, especially involving the use of byproducts containing chitin as the C/N-providing source.

### 3.2. Optimization of Culture Conditions for Exochitinase Production

**Figure 1:**
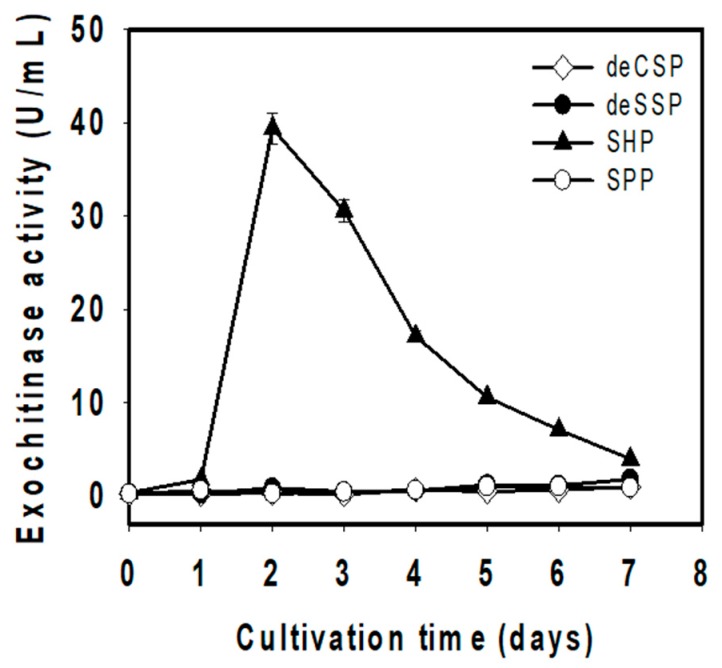
Effect of different chitinous byproducts on *S. speibonae* TKU048 exochitinase production. The medium was prepared by adding 1 g of each different fishery byproducts, including demineralized crab shell powder (deCSP), demineralized shrimp shell powder (deSSP), shrimp head powder (SHP), and squid pen powder (SPP) to 250 mL glass Erlenmeyer flasks containing 100 mL of basal salt medium. The cultivation conditions were conducted with 1% (*v*/*v*) of stock solution of *S*. *speibonae* TKU048, at an incubation temperature of 37 °C, and 150 rpm of agitation. An aliquot of culture (1 mL) was withdrawn every 24 h for testing exochitinase activity.

To explore the best C/N sources for TKU048 exochitinase production, four chitinous materials, SHP, SPP, deSSP, and deCSP, were added to the basal medium (0.05% MgSO_4_ and 0.1% K_2_HPO_4_) at a concentration of 1% (*w*/*v*) for cultivating *S. speibonae* TKU048. As shown in Figure 1, TKU048 exhibited the most exochitinase activity on SHP with 39.379 U/mL after 2 days of cultivation, while its activity was lower than 2 U/mL on other chitinous materials sources (SPP, deSSP, and deCSP). This result was different from the research of Tran et al. which showed that SPP was the most suitable for producing exochitinase by *S. thermocarboxydus* TKU045 (12.2 U/mL on SPP, approximately four-fold higher than 2.39 U/mL on SHP) [2]. This result suggests that the type of chitinous material has a significant effect on exochitinase production by *S. speibonae* TKU048, in which SHP, a common byproduct in the seafood processing industry, was demonstrated to be the best potential source. By achieving the highest exochitinase productivity in shorter cultivation times, SHP was selected as the best C/N source for cultivating *S. speibonae* TKU048.

The effects of other culture parameters on the production of TKU048 exochitinase, such as amount of SHP, incubation temperature, pH, and agitation rate, were also investigated. The results are summarized in Table 1. Following that, *S. speibonae* TKU048 was found to exhibit the highest exochitinase productivity on the medium containing 1.5% SHP with an initial pH of 6.0, incubation temperature of 37 °C, and shaking speed of 175 rpm. After 2 days of fermentation, the exochitinase activity of the optimized culture supernatant reached the maximum value at 45.668 U/mL. The exochitinase activity was approximately 45-fold higher than before optimization. This result indicated that the culture conditions for the production of *S. speibonae* TKU048 exochitinase by using only chitinous fishery byproducts to provide the source of C/N were successfully optimized. Until now, conversion of abundant and low-cost chitinous materials by microbial activity to produce bioactive compounds has received great attention [11,12,13,14,15,16,17,18,19,20,21,22,23,24,25,26,27,28,29,30,31]. Consequently, the current results could be promising in providing a novel beneficial application of shrimp heads—a chitin-containing byproduct from fishery processing—in producing exochitinase via *S. speibonae* TKU048.

### 3.3. Isolation of Exochitinase

**Figure 2:**
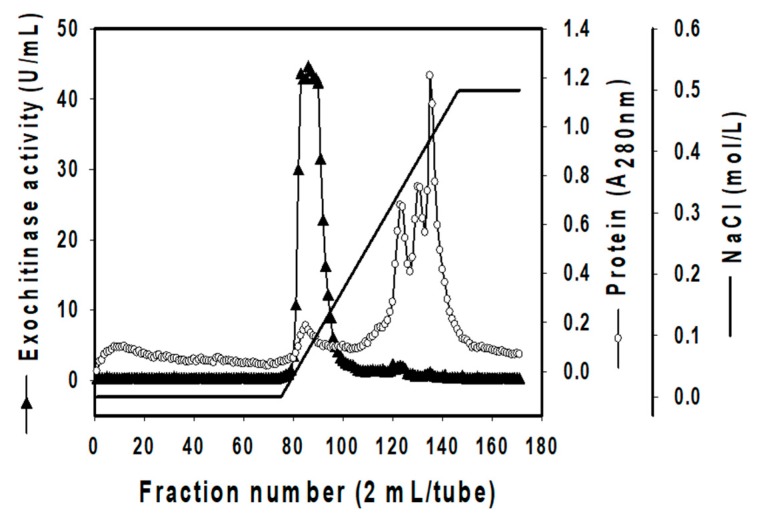
A typical ion-exchange chromatography profile of *S. speibonae* TKU048 exochitinase on Macro-Prep High Q. The elution was performed using Tris-HCl buffer system (20 mM, pH 7) with a NaCl gradient from 0 to 0.5 M and 2.5 mL of flow rate. Fifty microliters of each tube were withdrawn to test the exochitinase activity. The exochitinase activity fraction was found from tubes 80 to 91.

Since there are no previous reports of exochitinase from *S. speibonae*, it is necessary to investigate the characteristics of exochitinase from this species. To explore its characteristics for comparison with other reports, TKU048 exochitinase was isolated and purified by serial steps: ammonium sulfate precipitation, Macro-Prep High Q chromatography, and size exclusion chromatography on KW-802.5 column. The result is summarized in Table 2. Using ion-exchange chromatography, one peak showing exochitinase activity was found in eluted fractions 80–91 (Figure 2). These fractions were pooled for further purification by KW-802.5 column. Finally, 0.03 mg of TKU048 exochitinase was collected. The purification showed low yield recovery (0.1%), but high purity yield (376.3-fold). It also showed the strong specific activity result of the obtained enzyme (1.92 × 10^3^ U/mg), which was higher than in other reports [2,13,35,44,47,48,49]. This result indicates that the obtained enzyme not only showed high purity but also exhibited strong exochitinase activity.

**Figure 3:**
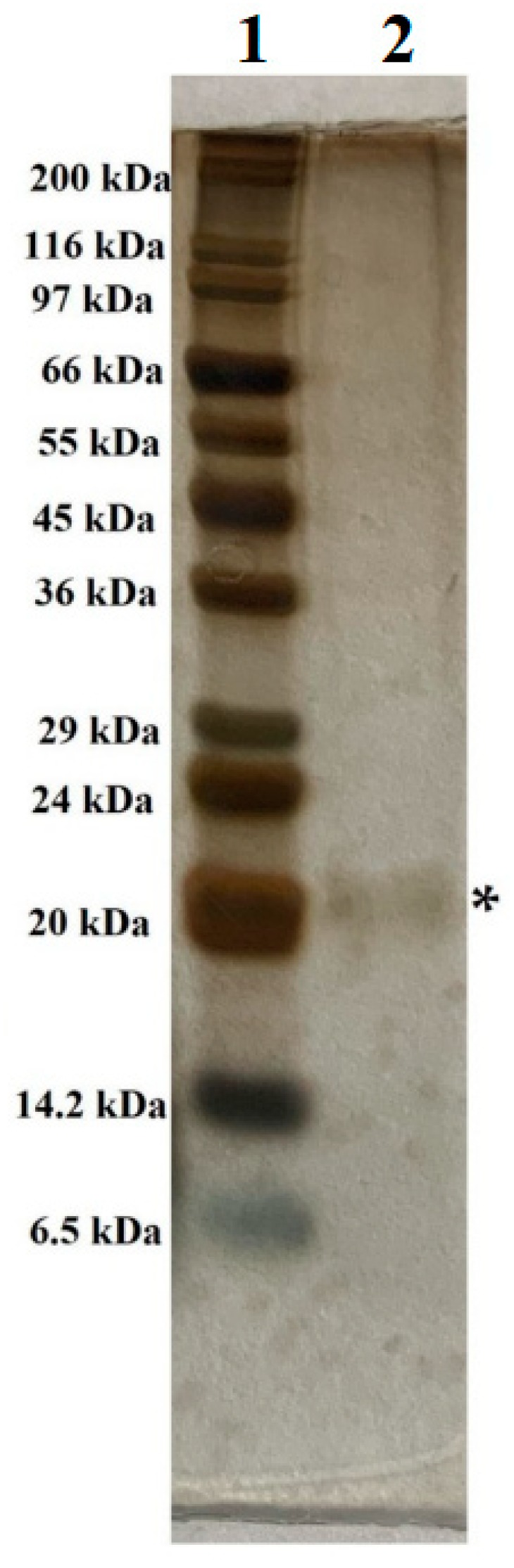
SDS-PAGE analysis of TKU048 exochitinase. 1, protein markers; 2, purified exochitinase after HPLC; *, location of purified TKU048 exochitinase.

According to Figure 3, the molecular weight of *S. speibonae* TKU048 was calculated to be approximately 21 kDa. The molecular weight of TKU048 exochitinase was consistent with *Streptomyces* sp. M-20 chitinase (20 kDa) [37], and smaller than that from other reports, for instance, *S. violaceusniger* XL-2 chitinase (28 kDa) [50], *Streptomyces* DA11 chitinase (34 kDa) [38], *S. griseus* MTCC 9723 chitinase (34 kDa) [40], *S. anulatus* CS242 chitinase (38 kDa) [51], *Streptomyces* CS147 chitinase (41 kDa) [52], *Streptomyces* sp. CS501 chitinase (43 kDa) [47], *S. halstedii* AJ-7 chitinase (55 kDa) [48], *S. violaceusniger* MTCC3959 chitinase (56.5 kDa) [41], *S. violascens* NRRL B2700 chitinase (65 kDa) [39], and *S. venezuelae* P10 (66 kDa) [49] with the exception of *S. thermocarboxydus* TKU045 chitinase (12.8 kDa) [2]. This indicates that *S. speibonae* TKU048 exochitinase is one of the smallest chitinases from the *Streptomyces* genus.

### 3.4. Effects of Temperature and pH on the Activity and Stability of TKU048 Activity

**Figure 4:**
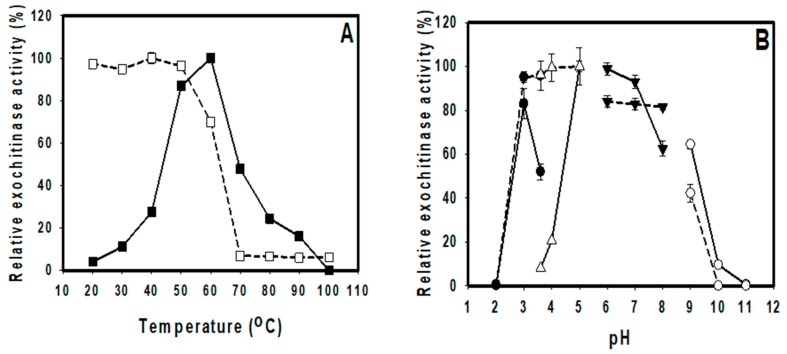
Effects of temperature (**A**) and pH (**B**) on the activity and stability TKU048 exochitinase: (—) optimum; (…) stability; (⚫) glycine HCl buffer; (△) sodium acetate buffer; (▼) sodium phosphate buffer; and (⚪) sodium bicarbonate–carbonate buffer.

A range of temperature from 20–100 °C was used to investigate the influence of temperature on TKU048 exochitinase activity. Figure 4 revealed that the optimal temperature of TKU048 exochitinase was 60 °C and its stability was up to 50 °C. However, at the optimal temperature, the enzyme still retained more than 60% of its activity. Among the chitinases from *Streptomyces* species, TKU048 exochitinase showed good thermal stability; most of them showed an optimal and stable temperature point similar to or lower than that of the TKU048 exochitinase [2] with some exceptions, such as *S. anulatus* CS242 chitinase (stability temperature was 60 °C) [51] or *S. thermoviolaceus* OPC-520 chitinase (optimal temperature was 70–80 °C) [53]. Thermal stability can benefit the application of chitinase in industrial uses.

The effects of pH on activity and stability of TKU048 chitinase were also studied herein. The optimal pH of TKU048 exochitinase was found at pH 5–7 (on sodium acetate and sodium phosphate buffer, respectively). However, the enzyme also exhibited another optimal pH point at pH 3 when using glycine HCl buffer with over than 80% activity, compared with its activity at pH 5. This suggests that the optimal pH of TKU048 exochitinase also depends on the buffer system. This result was different from most of the other reports of chitinase from other *Streptomyces* strains, which showed an optimal pH of 5–8 [37,38,39,40,41,47,48,50,51,52]; only several strains exhibited an optimal pH at a more acidic point, such as *S. thermocarboxydus* TKU045 (pH 4) [2] and *Streptomyces* sp. (pH 2 and 6) [54]. To determine the pH stability of *S. speibonae* TKU048 exochitinase, the enzyme was incubated at a range of pH from 2 to 11 using different buffer systems for 30 min, as mentioned above, and its residual activity was measured after adjusting the enzyme solution to pH 5. Since retaining over 80% of initial activity in pH range from 3–8, *S. speibonae* TKU048 exochitinase also exhibited good pH stability, especially under acidic conditions.

### 3.5. Substrate Specificity

Different kinds of substrates were used to investigate the specificity activity of TKU048 exochitinase. As shown in Table 3, TKU048 exochitinase expressed the best activity at 43.887 U/mL on *p*NPg (by *p*-nitrophenol method), followed by β-chitin powder (βCP) > colloidal chitosan (CCO) > colloidal chitin (CC) > water-soluble chitosan (WSC) > cellulose (by reducing sugar method). In addition, TKU048 exochitinase did not show activity on dextran and α-chitin powder (αCP). This result indicates that TKU048 chitinase specifically acted on the β-(1→4)-linkages and could hydrolyze different types of substrates, including chitin, chitosan, and cellulose. The different activity on αCP, and βCP suggest that the crystalline structure of chitin also affected the ability of TKU048 exochitinase. In addition, it was interesting that βCP was the most suitable substrate of TKU048 exochitinase, with the exception of *p*NPg. Generally, chitinases possess higher activity on colloidalchitin than on powder chitin [13,35,43,44]; however, some opposing results could be found, such as for chitinase and chitosanase from *B. cereus* TKU030 [21]. However, some chitinases have exhibited similar results; in producing chitin oligosaccharides or GlcNAc by enzymatic method, chitin must be pretreated with a strong acid, like concentrated HCl, in order to form colloidal chitin. This chemical process has several drawbacks, such as in releasing toxic wastewater and altering chitin’s structure. By showing the most activity on β-chitin powder, TKU048 exochitinase may have potential for the direct preparation of chitin oligosaccharides or GlcNAc from chitin powder.

### 3.6. Effects of Metal Ions

**Figure 5:**
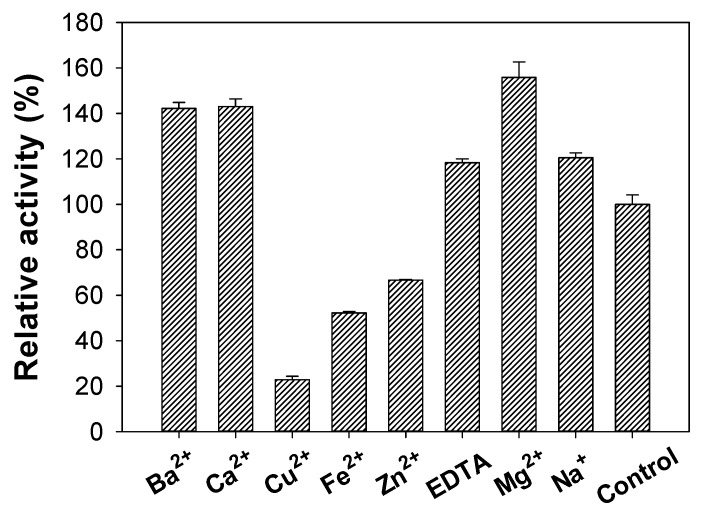
Effect of ion metals on the activity of TKU048 exochitinase. TKU048 exochitinase was pre-incubated with each of chemicals for 30 min. The activity of TKU048 exochitinase in the absence of treatment chemicals was used as a control to estimate relative activity (%).

As shown in Figure 5, *S. speibonae* TKU048 exochitinase was strongly inhibited by Cu^2+^ > Fe^2+^ > Zn^2+^. However, in the presence of Ba^2+^, Ca^2+^, Na^+^, Mg^2+^, and EDTA, TKU048 exochitinase possessed higher activity than that in the control. These results were markedly different from those of other reports [35,37,38,39].

### 3.7. Hydrolysis Mechanism

**Figure 6:**
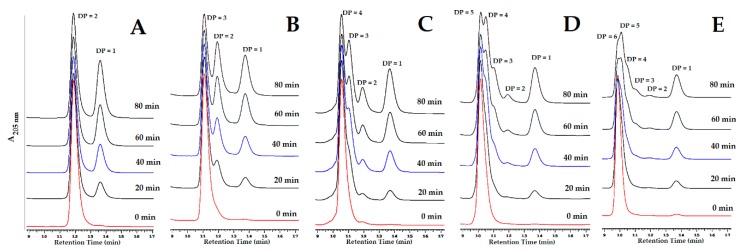
HPLC analysis of the hydrolysis products from (GlcNAc)_2–6_ by TKU048 exochitinase. **A**–**E**: (GlcNAc)_2_–(GlcNAc)_6_, respectively. The reaction was conducted by adding 500 µL substrate solution (0.5 mg/mL) with 500 µL enzyme solution (2 U, approximately) and incubated at 50 °C. Twenty microliters of sample was used for a single HPLC analysis.

To investigate the hydrolysis mechanism of TKU048 exochitinase, chitin oligosaccharides with degree of polymerization (DP) 2–6 were used as the substrates. As shown in Figure 6, TKU048 exochitinase could rapidly hydrolyze all chitin oligosaccharides (2–6 of DP) to release GlcNAc as the main product. This indicates that the obtained enzyme was an exochitinase. As far as we know, exochitinase was considered to separate chitobiase and *N*-acetyl-β-glucosaminidase. Due to its hydrolyzing abilities (GlcNAc)_2_ (Figure 6A), TKU048 exochitinase could initially be classified as an *N*-acetyl-β-glucosaminidase. Furthermore, the low rate of (GlcNAc)_2_ production from the hydrolysis of chitin oligosaccharides DP 3 to DP 6 (Figure 6C–E) revealed that the release of this dimer was not achieved by chitobiase activity. In Figure 6E, the minor (GlcNAc)_3_ liberated from the hydrolysis reaction of (GlcNAc)_6_ in the first 20 min indicates that TKU048 exochitinase did not express endochitinase or chitotriase activity. Taken together, the hydrolysis mechanism of TKU048 exochitinase was recognized as following *N*-acetyl-β-glucosaminidase activity, which catalyzes the hydrolysis reaction of chitin oligosaccharides at the end point to release GlcNAc. The hydrolysis mechanism of TKU048 exochitinase was different from the descriptions in several reports; for instance, *Chitinolyticbacter meiyuanensis* SYBC-H1 strain produced a chitinase (CmChi1) which possessed both exochitinase and endochitinase abilities and poor *N*-acetyl-β-glucosaminidase activity [43], *Pb*Chi70 produced by *P. barengoltzii* showed only exochitinase activity [55], PbChi74 produced by *P. barengoltzii* possessed two chitinolytic activities (exochitinase and *N*-acetyl-β-glucosaminidase) but lacked endochitinase activity [44], and exo-Chi O1 from *S. olivaceoviridis* was demonstrated to be an exochitinase that catalyzed chitin to release chitin oligosaccharide with DP 2 as the major product [45].

### 3.8. Evaluation of GlcNAc Production by TKU048 Exochitinase

**Figure 7:**
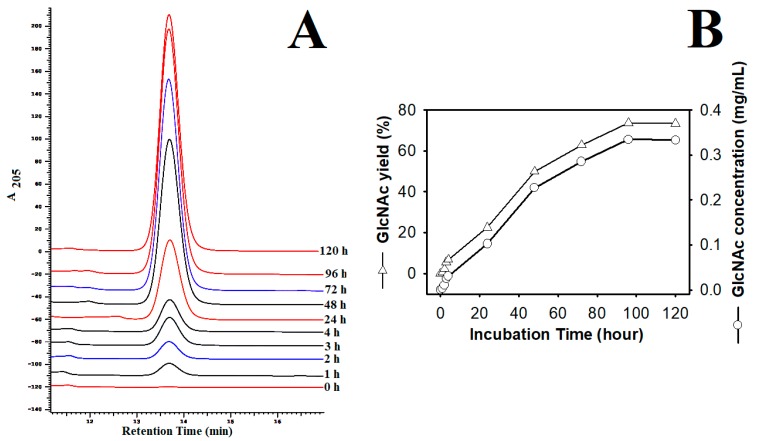
The hydrolysis of β-chitin powder by TKU048 exochitinase: **A**: HPLC analysis of chitin hydrolysis pattern; **B**, the time course of the chitin hydrolysis of chitin.

Since β-chitin powder was demonstrated to be a suitable substrate for TKU048 exochitinase, this material was chosen for GlcNAc production. The hydrolysis reaction was performed in sodium acetate buffer (50 mM, pH 5) with 0.455 mg/mL of β-chitin powder concentration and 2 U/mL of TKU048 exochitinase (measured by *p*-nitrophenol reference, approximately) on an incubator (150 rpm, 50 °C). As shown in Figure 7A, the peaks indicating GlcNAc appeared at the retention time of 13.69 min, and the maximum value was observed after 96 h of incubation time. The area of GlcNAc peaks was then used to calculate GlcNAc concentration and GlcNAc production yield. It was found that GlcNAc concentration and GlcNAc yield increased over time (Figure 7B). Finally, 0.335 mg/mL of GlcNAc could be obtained from 0.455 mg/mL of β-chitin powder with a yield of 73.64% in 96 h. Several reports show that the hydrolysis of chitin by chitinases observed the GlcNAc concentration in the range of 9.8–39.3 µg/mL [43,56]. The higher GlcNAc may have an inhibitory effect on the activity of chitinases [56]. Consequently, it indicated that TKU048 exochitinase may be suitable for GlcNAc production in a higher concentration of this product.

## 4. Conclusions

One of the most important applications of chitinase is its use in hydrolyzing chitin/chitosan to produce bioactive chitooligosaccharides and GlcNAc. In the current study, exochitinase production was reported on a novel bacterial strain, *S. speibonae* TKU048, by using shrimp heads, a low-cost chitinous material, as the sole C/N source. *S. speibonae* TKU048 exochitinase was purified with high specific activity (1.92 × 10^3^ U/mg) and had a molecular mass of 21 kDa. The enzyme also showed valuable properties such as thermal stability, optimal acidic pH, and degradable *β*-chitin powder. In addition, the hydrolysis mechanism of TKU048 was investigated, which mainly followed *N*-acetyl-*β*-glucosaminidase activity. The result also indicated that TKU048 exochitinase could hydrolyze *β*-chitin powder to release GlcNAc at high concentrations. The excellent characteristics of *S. speibonae* TKU048 may give it great potential in GlcNAc production.

## Figures and Tables

**Table 1:** Comparison of culture conditions before and after optimization.

Compared Factors	Before Optimization	After Optimization
Type of chitinous byproduct	SPP	SHP
Amount of C/N source (%)	1	1.5
Cultivation temperature (°C)	37	37
Initial pH	7.8	6.0
Shaking speed (rpm)	150	175
Incubation time (day)	3	2
Exochitinase activity (U/mL)	1.001	45.668

**Table 2:** Purification of the exochitinase from *S. speibonae* TKU048.

Steps	Total		Specific Activity (U/mg)	Purification Fold	Recovery Activity Yield (%)
	Protein (mg)	Activity (U)			
Culture supernatant	9.24 × 10^3^	4.71 × 10^4^	5.10	1.0	100.0
(NH_4_)_2_SO_4_ ppt.	2.47 × 10^3^	1.56 × 10^4^	6.33	1.2	33.2
Macro-Prep High Q column	1.35	1.45 × 10^3^	1.08 × 10^3^	211.3	3.1
KW-802.5 column	0.03	48.09	1.92 × 10^3^	376.3	0.1

**Table 3:** Substrate specificity of TKU048 exochitinase.

Substrate *	Chitinolytic Activity (U/mL)
*p*NPg	43.887 ± 0.698
Dextran	0
WSC	0.258 ± 0.008
βCP	0.406 ± 0.003
CC	0.319 ± 0.002
Cellulose powder	0.218 ± 0.024
αCP	0
CCO	0.341 ± 0.034

* WSC: water-soluble chitosan; βCP: β chitin powder; CC: colloidal chitin; αCP: α chitin powder; CCO: colloidal chitisan.
